# Zinc and Copper Enhance Cucumber Tolerance to Fusaric Acid by Mediating Its Distribution and Toxicity and Modifying the Antioxidant System

**DOI:** 10.3390/ijms21093370

**Published:** 2020-05-10

**Authors:** Ruirui Wang, Jian Huang, Aichen Liang, Ying Wang, Luis Alejandro Jose Mur, Min Wang, Shiwei Guo

**Affiliations:** 1 Jiangsu Provincial Key Lab for Organic Solid Waste Utilization, National Engineering Research Center for Organic-based Fertilizers, Jiangsu Collaborative Innovation Center for Solid Organic Waste Resource Utilization, Nanjing Agricultural University, Nanjing 210095, China; 2017203055@njau.edu.cn (R.W.); 2019103100@njau.edu.cn (J.H.); 2018103103@njau.edu.cn (A.L.); 2017103098@njau.edu.cn (Y.W.); sguo@njau.edu.cn (S.G.); 2 Institute of Biological, Environmental and Rural Sciences, Aberystwyth University, Aberystwyth SY23 3DA, UK; lum@aber.ac.uk

**Keywords:** fusaric acid, zinc, copper, distribution, mitigation, oxidative stress

## Abstract

Fusaric acid (FA), the fungal toxin produced by *Fusarium oxysporum*, plays a predominant role in the virulence and symptom development of Fusarium wilt disease. As mineral nutrients can be protective agents against Fusarium wilt, hydroponic experiments employing zinc (Zn) and copper (Cu) followed by FA treatment were conducted in a glasshouse. FA exhibited strong phytotoxicity on cucumber plants, which was reversed by the addition of Zn or Cu. Thus, Zn or Cu dramatically reduced the wilt index, alleviated the leaf or root cell membrane injury and mitigated against the FA inhibition of plant growth and photosynthesis. Cucumber plants grown with Zn exhibited decreased FA transportation to shoots and a 17% increase in toxicity mitigation and showed minimal hydrogen peroxide, lipid peroxidation level with the increased of antioxidant enzymes activity in both roots and leaves. Cucumber grown with additional Cu absorbed less FA but showed more toxicity mitigation at 20% compared to with additional Zn and exhibited decreased hydrogen peroxide level and increased antioxidant enzymes activity. Thus, adding Zn or Cu can decrease the toxicity of the FA by affecting the absorption or transportation of the FA in plants and mitigate toxicity possibly through chelation. Zn and Cu modify the antioxidant system to scavenge hydrogen peroxide for suppressing FA induction of oxidative damage. Our experiments could provide a theoretical basis for the direct application of micro-fertilizer as protective agents in farming.

## 1. Introduction

The soil-borne fungus *Fusarium oxysporum* has been identified as the fifth most important fungus in plant pathology as it causes vascular wilt disease in more than 100 different crops [1]. Fusaric acid (FA), a potent host non-specific toxin produced by *Fusarium* species, is the main cause of the development of Fusarium wilt disease [2,3,4,5]. FA has negative effects on cell integrity [6,7] and function [8]. FA, a cell death inducer, produces an enormous oxidative burst, which is evident from an enhancement in lipid peroxidation, causing cellular dysfunction. FA alters membrane permeability, inhibits ATP synthesis and disturbs the water balance through uncontrolled water loss from injured cells [9,10,11,12]. FA causes the reduction of root elongation in *Arabidopsis* and date palm [13,14] and root cell dysfunction in tomato and cucumber seedlings [15,16]. Further, these all confirm the major and even decisive role of FA in the disease progression. Currently, Fusarium wilt is controlled through pesticides, chemical soil fumigation and use of resistant cultivars. Other biological strategies include the grafting of cucumber with resistant rootstocks, such as bottle gourd, as commonly used in Korea [17]. Considering the environmental effects of synthetic pesticides, their high costs and challenging application procedure using nutritional regulation as protective strategies represents worthwhile and promising alternative.

Micronutrients are essential to plants and can influence disease tolerance or resistance in plants. Mechanistically, this may involve direct toxicity but also the triggering of organic defenses and chelation; features that are grouped in the elemental defense hypothesis [18,19,20]. Direct toxicity metal ions on pathogens or herbivores is very well established [21,22,23]. Plants naturally absorb high concentrations of metals from the substrate as a self-defense mechanism directly against pathogens and herbivores [24], such as iron (Fe) [25], zinc (Zn) [23,26] and copper (Cu) [23,26,27]. Exceptionally high Zn concentrations directly suppress powdery mildew disease symptoms of hyperaccumulator plants *Thlaspi caerulescens* or protect *Noccaea caerulescens* against *Alternaria brassicicola* by limiting pathogen colonization [28,29,30]. Manganese (Mn) application can effectively control the occurrence of downy mildew, powdery mildew, brown spot disease and other diseases [31,32]. Unsurprisingly, farmers use metal-containing compounds as leaf sprays or soil amendments in phytosanitary treatments.

Metal ions can evoke defense reactions and can sometimes drive pathogen resistance of non-hyperaccumulator plants [18,19,20,33]. Hence, the metal ion-elicited defense could be as effective in a non-hyperaccumulator plant species [19]. The metal ions could elicit defense reactions through signaling pathway or by plant fortification. For example, adequate Zn levels can trigger signaling mechanisms, which induce the JA/ET signaling pathway leading to camelexin accumulation and improved resistance to *Alternaria brassicicola* in *Arabidopsis thaliana* [34]. However, constitutively high concentrations of salicylate found in the hyperaccumulator species of *Thlaspi* might render these plants insensitive to pathogen-induced signals that are required for defense induction [35]. Metal-induced reactive oxygen species (ROS) can be responsible for the induction to mechanical defenses, defense signals, the pathogen-resistance-related defense genes and the synthesis of defense-related secondary metabolites [36,37]. Besides direct toxicity of the element to pathogens and herbivores, alone or in combination with organic defenses, several studies have suggested that the high phytotoxicity of FA is most likely due to the ability to chelate different metal ions [38,39], such as Fe, Cu, Zn or Mn [10,38,40,41,42]. The phytotoxic effect of FA produced by *Fusarium oxysporum* on tomato plants has been shown to be influenced by chelation of Cu, Fe or Zn *in planta* [43].

Previous studies have focused on identifying defense mechanism via the elemental defense hypothesis; these have been complicated if considering the possible interactions of the metal with both the host and the pathogen. Little research has focused on assessing the effect of metal ions on pathogen produced secondary metabolites linked to virulence. In the present study, hydroponic experiments were conducted to investigate the possibility of a direct elemental defense against FA. It was hypothesized that metal could play a vital role in FA-induced oxidative stress. We also monitored the fate of FA, including its transportation and distribution *in planta* and the toxicity mitigation in vitro. We demonstrate that Cu/Zn treatments represent a valid means to suppress FA effect on plants, although each metal can act via different mechanisms.

## 2. Results

### 2.1. FA Toxicity in Cucumber Plants is Reversed by the Exogenous Addition of Zn and Cu

To test the link between FA phytotoxicity and metal ions, three-week-old cucumber plants were hydroponically fed with solutions containing either Fe, Mn, Cu or Zn for 24 h before adding 2 µg mL^−1^ FA. FA treated plants exhibited progressive wilt of the stem and new leaf, with necrotic roots which came to be seen in the leaves and finally resulting in the wilting of the entire plant (CK + FA). Seedlings effectively relieved the symptoms of wilt with the treatments of high concentrations of Zn or Cu.

**Figure 1:**
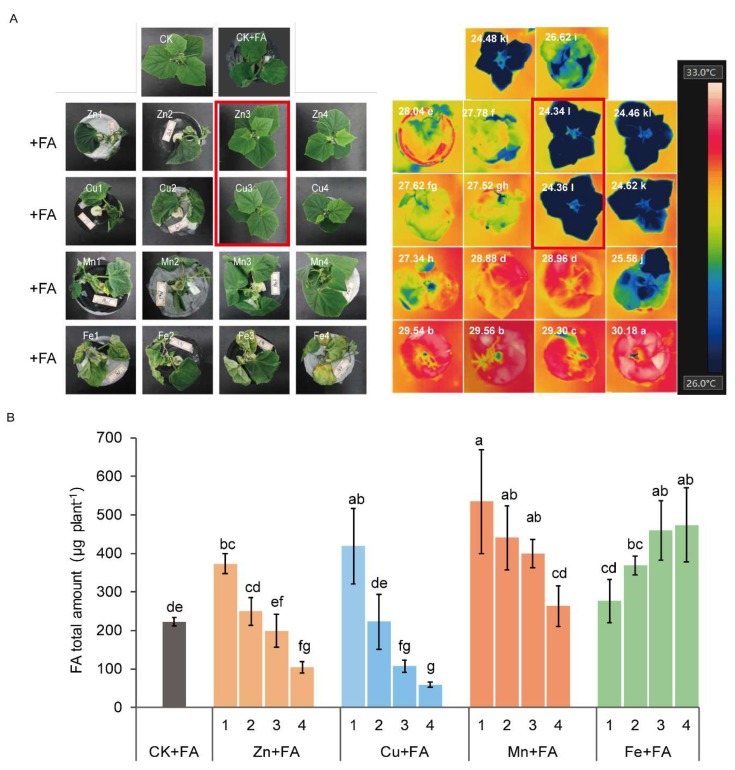
Effects of Fe, Mn, Cu and Zn on cucumber plants under fusaric acid (FA) supply. (**A**) 3-week-old seedlings were cultured with Hoagland solutions containing 2 µg mL^−1^ FA and different concentrations of Fe, Mn, Cu or Zn. CK, control plants; CK + FA, control plants after FA treatment. Each ion consists of four concentration series, denoted by 1, 2, 3, 4; Zn concentrations were 0, 9.6, 48 and 144 µM; Cu concentrations were 0, 2, 20 and 40 µM; Mn concentrations were 0, 57, 114 and 570 µM; Fe concentrations were 0, 89.5, 179 and 358 µM. The two treatments in the red box were selected for the following experiments. Thermal images of cucumber plants under light conditions after different treatments were shown on the right. The treatments directly correspond to the left. The temperature quantification results represent the means of four replicates (°C). (**B**) FA total amount in the plant with different treatments. The letters in the thermal image on the right side of figure (**A**) indicate statistically significant differences (*p* < 0.05) among all treatments (a total of 18 treatments). The letters above the bars of the histogram in figure (**B**) indicate statistically significant differences (*p* < 0.05) among all different treatments (a total of 17 treatments) and bars represent standard deviation with four replications.

The overall temperature of leaves in plants with high concentrations of Zn or Cu was clearly lower than that of control plants with FA treatment (Figure 1A). The total content of FA significantly decreased when the Zn or Cu concentration increased, whilst the content of FA in plants was significantly higher, regardless of the concentration of Mn or Fe (Figure 1B). Those solutions with the highest concentrations of Zn or Cu (Zn4, Cu4) had a slightly toxic effect on plants (Figure 1A). As a result, in the following experiments, 48 µM Zn (Zn3) and 20 µM Cu (Cu3) were used for all subsequently Cu and Zn treatments and denoted by Zn + FA and Cu + FA respectively. 0.96 µM Zn and 0.45 µM Cu were used for control plants (CK) and control plants after FA treatment (CK + FA).

In the absence or presence of the FA, no significant differences in plant growth and health were observed between plants supplemented Zn or Cu and control plants, referring to the preliminary experimental exploration phase (Figure S1). The next experiment focused on the effect of metal ions on FA. FA treatment results in a decreased biomass tendency, without growth promotion (Table 1). Within the time frame of the experiment, neither toxicity symptoms nor growth inhibition was observed in plants supplied with Zn or Cu. The leaf net photosynthetic rate of plants with FA treatment was approximately 19% and 23% lower than Zn + FA and Cu + FA plants. Similarly, stomatal conductance, intercellular CO_2_ concentrations and transpiration rates of plants after FA treatment were significantly lower compared to other treatments (Table 1). The reduced stomatal conductance after FA treatment was linked to a lower leaf transpiration rate (E), but the ratio of the transpiration rate to stomatal conductance (E/g_s_) was markedly increased. These data indicate that water was lost from a non-stomatal pathway in control plants after FA treatment, but the addition of Zn and Cu eased this effect. Zn or Cu treatment also showed enhanced tolerance against FA as indicated by a clear reduction in wilt index score (Table 1).

### 2.2. Additional Zn or Cu Improves the Tolerance of Cucumber to FA by Mediating Its Distribution

**Figure 2:**
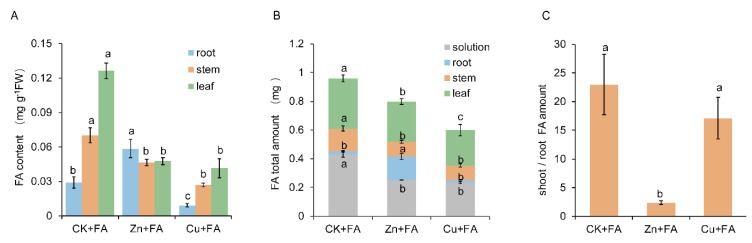
Zinc and copper had different effects on fusaric acid (FA) absorption and distribution in plants. (**A**) FA content in the root, stem and leaf of differentially treated cucumber plants. (**B**) The total amount of FA in plant tissues and nutrient solutions. (**C**) The ratio of shoot to root FA content. Bars represent standard deviation with four replications each. Different letters indicate significant differences (*p* < 0.05, Duncan’s multiple range test) of the same tissue with different treatments.

To investigate the tolerance mechanisms of plants conferred by Zn and Cu, the distribution patterns of FA distribution were measured in the roots, stems and leaves of all cucumber plants. FA mostly accumulated in the roots of plants grown with Zn, accounting for 30% of the total absorption (Figure 2A,B). Zn supply led a down-regulation of the FA shoot/root ratio of 11 times compared to plants treated only with FA (Figure 2C). In contrast, Cu + FA and FA-treated plants exhibited most FA in the shoots (on average 95%) (Figure 2B). Nevertheless, the content or total amount of FA was the lowest in Cu + FA-treated plants, whether root, stem or leaf (Figure 2A,B). These results indicated that Zn supply relatively reduced FA transportation to the shoot and Cu supply relatively reduced FA uptake from the hydroponic solution, which contributed to the increased tolerance of plants to FA.

**Figure 3:**
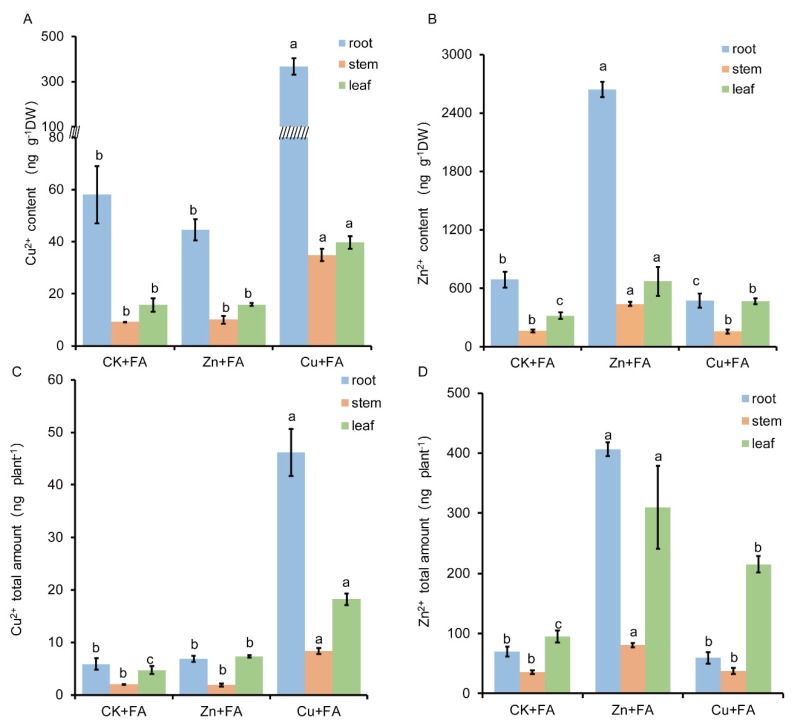
Relationship between Cu^2+^ and Zn^2+^ distribution and the FA distribution. Cu^2+^ or Zn^2+^ content (**A**,**B**) and amount (**C**,**D**) of cucumber plant issues treated with fusaric acid (FA). Bars represent the standard deviation of three replicated each. Different letters indicate significant differences (*p* < 0.05, Duncan’s multiple range test) of the same tissue among three treatments.

To investigate whether the content of Zn or Cu in tissues is associated with decreased transportation or absorption of FA, the tissue metal content of plants was determined using inductively coupled plasma mass spectrometry (ICP-MS). A significant increase in the tissue Zn^2+^ or Cu^2+^ content or total amount was also observed in plants grown with Zn or Cu supply, especially in roots. There was no significant difference between the tissue Zn or Cu content or total amount of the other two treatments (Figure 3). The Cu^2+^ concentration of tissues increased significantly, and correspondingly, the content of FA of tissues was the lowest (Figure 2A,B and Figure 3A,C). That’s not the case for Zn (Figure 2A,B).

### 2.3. FA is Detoxified through Direct Reaction with Zn or Cu

**Figure 4:**
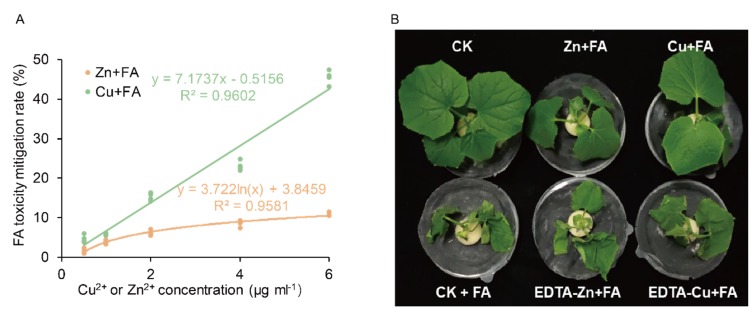
Ions mitigated toxicity of FA in vitro. (**A**) CuSO_4_ and ZnSO_4_ as resources of Cu and Zn were mixed in the FA aqueous solution (2 µg mL^−1^) for 24 h. Zn or Cu concentrations were 8, 16, 32, 64 and 96 µM. (**B**) CuSO_4_ aqueous solution (200 µM) or ZnSO_4_ aqueous solution (480 µM) or EDTA-Cu (200 µM) or EDTA-Zn aqueous (480 µM) solution were mixed with FA (20 µg mL^−1^) in vitro for 24 h and then seedlings were placed into each respective solution. Plant symptoms were imaged after a further 3 days.

The FA remaining in the nutrient solution after harvesting plants was determined and used to derive a value for the total FA content (plant + solution). The total FA content of Zn treated plants was reduced by 17%, but with Cu treatments, this was reduced by 37% (Figure 2B). To assess whether this reduction could reflect direct chemical impacts on Cu or Zn on FA rather than effects arising from metal treated plants, a series of plant-free tests were undertaken. Various concentrations of Cu and Zn were mixed with an aqueous solution of FA (2 µg mL^−1^), and the concentration of FA was determined after 24 h. There was a significant positive correlation between the FA toxicity mitigation and the Zn^2+^ or Cu^2+^ content (Figure 4A), which was consistent with the *in planta* distribution and content of Cu and FA (Figure 2A, Figure 3A). Compared with Zn^2+^, Cu^2+^ had a stronger mitigation property.

As the products of this detoxification mechanism which could still be toxic, it’s *in planta* effects were assessed. Aqueous solutions of CuSO_4_, EDTA-Cu, ZnSO_4_ or EDTA-Zn were mixed with a higher concentration of FA (20 µg mL^−1^) in vitro for 24 h, and then seedlings were placed into each solution. Seedlings placed into EDTA-Cu + FA or EDTA-Zn + FA solutions exhibited equivalent wilting as saw in the CK + FA solution (Figure 4B). The seedlings placed in the Cu + FA solution were more vibrant than that in the Zn + FA solution, which was consistent with in vitro results (Figure 4A). The results showed that Cu alleviated toxicity in part through the destruction of FA to unknown products, possibly via chelation.

### 2.4. Additional Zn or Cu Alleviates the Oxidative Stress Caused by FA

**Figure 5:**
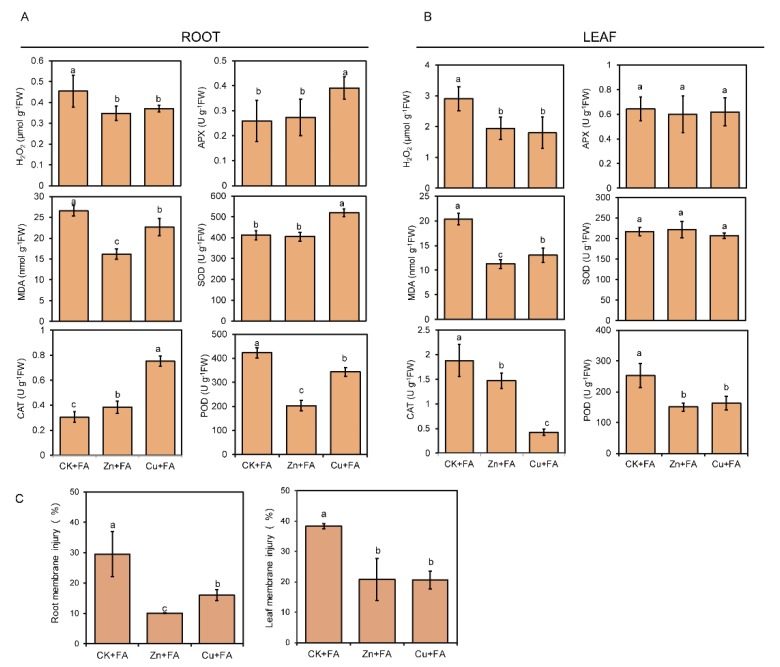
Effects of Zn or Cu on FA elicited ROS and cell membrane injury mitigation in leaf and root. The root (**A**) and leaf (**B**) H_2_O_2_, MDA and antioxidant enzyme activity levels were determined after treatment with FA for 72 h. (**C**) Relative membrane injury in the root and leaf. Bars represent standard deviation with four replications each. Different letters above bars indicate significant differences (*p* < 0.05, Duncan’s multiple range test) among different treatments.

The oxidative damage and membrane lipid peroxidation were analyzed in terms of the contents of hydrogen peroxide (H_2_O_2_) and malondialdehyde (MDA). Membrane injury assessments in the leaves and roots also indicated a protective effect of the Zn or Cu against FA. Consistent with the strong membrane injury observed in the roots and leaves treated with FA (Figure 5C), FA treatment enhanced H_2_O_2_ release and MDA content in both roots and leaves (Figure 5A,B). In contrast, H_2_O_2_ content and MDA content significantly decreased in both roots and leaves of plants supplied with Zn or Cu (Figure 5A,B).And this subtraction is mainly caused by Zn and Cu, because the effect of Zn or Cu alone on H_2_O_2_ release and MDA content is significantly less than that of FA alone (Figure S2). Compared with the control, Zn treatment alone increased H_2_O_2_ release and MDA content by 29% and 38%, respectively; Cu treatment increased H_2_O_2_ release and MDA content by 27% and 53%, and FA treatment increased H_2_O_2_ release and MDA content by 112% and 147%, respectively (Figure S2, Figure 5).The enzymatic antioxidant defense system including superoxide dismutase (SOD), catalase (CAT), peroxidase (POD) and ascorbate peroxidase (APX) is important for the plant to cope with ROS bursts during FA treatment. Compared with the control, Zn or Cu treatment alone significantly increased the activity of CAT by 85% and 150% in root and 52% and 28% in leaf, respectively (Figure S2). The antioxidant enzyme showed different responses to FA among different treatments. Compared with the CK + FA, Cu supply significantly increased the activity of CAT by 147%, the activity of APX by 50% and the activity of SOD by 26% in roots. A slight increase in CAT activity was observed in the roots of plants supply with Zn (Figure 5A). However, Cu and Zn supply led a down-regulation of the activities of POD in both roots and leaves, as well as no difference in the activities of APX and SOD was found in the leaves of Zn or Cu treated plant (Figure 5B). Similarly, the increase in the activity of antioxidant enzymes is mainly caused by Zn or Cu (Figure S2).

## 3. Discussion

Several studies have established the phytotoxic activity of FA and its role in the induction of wilt symptoms in plants [44]. For example, the addition of FA at concentrations above 0.5 mM to the culture medium significantly affected the growth of barley callus [45]. Our previous study confirmed that cucumber seedlings develop typical wilt symptoms when their roots are exposed to FA. The wilting was mainly due to uncontrolled non-stomatal water loss from damaged cells caused by FA [12]. In this work, FA was shown to reduce the stomatal conductance and transpiration rates of cucumber plants but increase the ratio of the transpiration rate to stomatal conductance (E/g_s_) (Table 1). In exploring the effect of metal on FA-effects, the additional Zn and Cu decreased E/g_s_ and relieved the damage to the cell membrane of leaves (Table 1, Figure 5C). These effects were mainly due to the concentration of shoot FA not reaching the toxic dose. We show that FA was detoxified either within the root and the surrounding root milieu i.e., the nutrient solution.

Current knowledge suggests that plants absorb high concentrations of metals as a self-defense mechanism against pathogens and herbivores to act via direct toxicity, triggering organic defenses or chelation reactions [18,19]. For hyperaccumulator plants, high levels of metal accumulation in shoot tissues of metal-tolerant hyperaccumulators can substitute for defects in common biochemical defenses or biotic stress signaling and protect these plants against disease [28,46,47]. Here, we show that the addition of Cu^2+^ or Zn^2+^ to FA-treated cucumber plants (a non-hyperaccumulator plant species), significantly reduces wilting (Figure 1, Table 1). On measuring the concentration of Zn and Cu in plant issues these were increased to about 4-fold (4 µg g^−1^) or 7-fold (0.5 µg g^−1^) respectively over controls (Figure 3). These Zn or Cu concentrations were within the common range considering agricultural food security [48,49]. Further, these Zn or Cu concentration were far below those seen with hyperaccumulator plants and have no direct toxicity to the pathogen. One study showed that trace amounts of Cu^2+^ and Zn^2+^ (20 µg mL^−1^) suppressed FA production when the fungus (here *F. oxysporum* f. sp. *ciceri*) was cultured in liquid medium [50].

FA was detected in the roots, stems and leaves of FA-treated cucumber plants, indicating that FA can be transported and distributed throughout the entire plant (Figure 2). However, cucumber grew with additional Cu absorbed and accumulated less FA compared to Zn (Figure 2). If only considering the role of the root, these differences in Cu/Zn effects could reflect differential impacts on root morphology, physiological and biochemistry to affect the distribution of FA. FA, behaving like a weak acid, passively permeates through the cell in uncharged form and accumulates in the cytosol in charged form, which could be bind to walls and membranes [51]. Therefore the main mechanisms of fusaric acid phytotoxicity have been suggested to be disruption of plasma membrane transport processes [52]. Vacuoles, cell walls and subepidermal intercellular space are main sites of storage of Zn; epidermal cell vacuoles have been reported to be the preferential site of Zn transport [53]. In the root, Zn is also mostly distributed in vacuoles [54]. There are many binding sites for Zn within the membranes, particularly in the interior sites of membranes [52]. Vacuolar sequestration capacity, xylem transport and phloem remobilization contribute essentially to metal distribution and long-distance metal transport in plants [55,56,57]. Meanwhile, compared with Cu, Zn is more transported to the shoot (Figure 3B,D). Therefore, the competition of binding sites or potential balance of the plasma membrane resulted in the accumulation of FA mainly in the root of Zn-treated plants, without transporting to the shoot (Figure 2, Figure 3D). Whilst Cu is mainly found in the cell walls [58]. Such differences could have important but need to be further assessed.

Moreover, metal tolerance in the plant is chelation of metal ions into complexes to reduce their toxicity [59]. We suggest that in addition to plant-based responses, it is probable that chemical chelation between FA and Cu or Zn represents a toxicity mitigation mechanism. This is based on studies that have suggested that FA has the ability to chelate different metal ions, such as Fe, Cu, Zn or Mn [10,38,39,40,42,60]. In our experiment, each plant received the same amount of FA. Therefore, by measuring the FA contents in plants and what was left in the nutrient solution could indicate how much could be lost through toxicity mitigation by such mechanisms as chelation. In previous experiments, the highest concentrations of Fe and Mn had a slightly toxic effect on plants. It was found that the four concentration gradients of Fe and Mn did not alleviate the toxicity of FA, which may be due to the fact that the ion concentration of Fe and Mn was not enough to chelate the toxicity of FA. Compared with other ions, Fe is easier to be chelated [38]. One study showed the chelation mechanism occurs inside the plant [43] and we did indeed show that the addition of Cu or Zn in FA-treated plants significantly reduces wilting and that both Zn or Cu is mainly accumulated in the root of plants (Figure 2). However, in a crucial in vitro, plant-free experiment, either Cu or Zn alone was able to lower FA concentrations (Figure 4A). Even if the concentration of ions and FA were increased simultaneously, the mitigation effect was also obvious. If the available Cu/Zn was sequestered using EDTA (i.e., EDTA-Cu or EDTA-Zn) the loss in FA was reduced (Figure 4B). This demonstrated the importance of free Cu/Zn, possibly to be chelated by FA. Further, the lack of any toxic products of Cu/Zn + FA as seedlings grown in the post-reaction solution failed to show toxicity. This stated, we recognize that metal-chelating mechanisms occur outside or inside plants, therefore, it is relevant that some studies showed increase FA toxicity with chelation, inhibiting metal-containing oxidative enzymes [61], altering the platelet aggregation process [62] and malformation [42]. As a result, the *in planta* relevance of our plant free experiments could be questioned. More importantly, as transition metal, Cu/Zn could be acting via redox-active mechanisms to reduce FA concentrations rather than via chelation. We note that our experiments would not be able to distinguish between chelation or redox destruction of FA or indeed if both were involved.

When cucumber plants were exposed to 50 times higher metal concentrations than controls, they did not show any growth inhibition but alleviated the inhibition of plant growth and photosynthesis by FA (Table 1, Figure 1). This led us to hypothesize that, besides direct toxicity, metal-triggered defenses act to improve plant tolerance. Metal is the most toxic as free ions [63] and plants may tolerate elevated intracellular metal concentrations by mechanisms which include redox-related compounds, enzymes, metal-binding ligands or proteins [28,64]. Such changes may induce mechanical fortification (cell wall lignification) and trigger defense signals [36,37]. Zn plays an irreplaceable role in cell metabolism and partial function of many membrane proteins [65,66]. Furthermore, Zinc, representing an excellent protective agent, contributes to plant tolerance to environmental stress by scavenging ROS [67] or inhibiting the production of highly toxic hydroxyl radicals (OH) [68]. FA induces cell death with an enormous oxidative burst during which large quantities of ROS like H_2_O_2_ was generated [69]. In consequence, plants supply with Zn showed minimal H_2_O_2_ and MDA content with minimal increased of CAT, APX, SOD and POD activity in roots (Figure 5B); supply Zn suppressed oxidative burst. Copper is a redox-active transition element with roles in photosynthesis, respiration, C and N metabolism, and protection against oxidative stress [70]. In higher plants, CuZn-SOD is the most abundant SOD, Cu is also indirectly required for the CuZn-SOD. In agreement with this, supply Cu increased antioxidant enzymes activity to scavenge the H_2_O_2_ (Figure 5A). The activity of CAT in the leaves of plants supplied with Cu or Zn was significantly lower than that of the plants only treated by FA, possibly because there was no oxidative stress in the leaves. Moreover, the POD activity in leaves is relatively low. Zn or Cu pretreatment caused oxidative stress and increased the activity of antioxidant enzymes in plants at the same time (Figure S2). Therefore, when the plant was subjected to FA stress again, the oxidative stress caused by FA would be alleviated (Figure 5). The antioxidant defense machinery is of paramount importance in protecting plants against oxidative damages [71,72]. These different roles may contribute to the different performances of plants roots given high Zn or Cu and tolerance to FA (Figure 5C).

**Figure 6:**
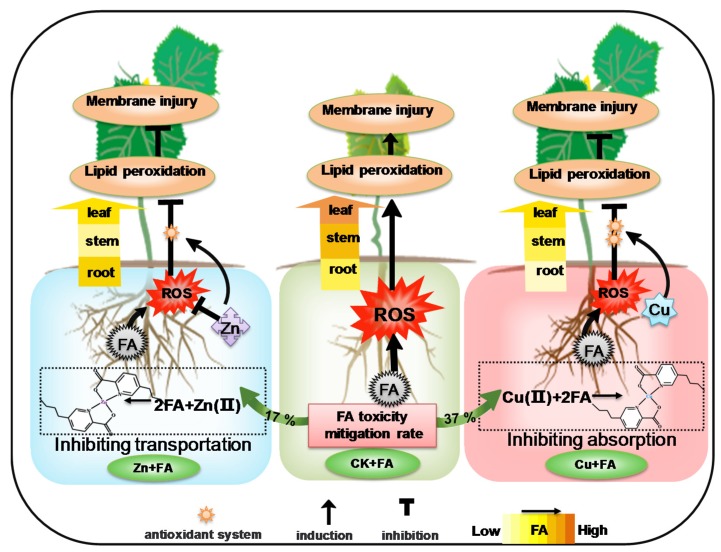
Ions enhance the tolerance of cucumber to fusaric acid by different mechanisms. Changing transportation (Zn) or absorption (Cu) and chelation reaction plays pivotal roles in toxicity mitigation of FA. Zn or Cu alleviates the ROS damage (lipid peroxidation, membrane injury) of FA to cucumber plants by modification in antioxidant enzymes system. The mitigation reaction, which could involve chelation, occurs in both Zn + FA and Cu + FA treatments. The arrow in green indicates the mitigation rate compared to the control. Arrows in yellow represent the transportation of FA to the ground under different treatments. Different degrees of yellow represent the FA content.

Based on our work, we have developed a mechanistic model of metal ions defense against FA produced by *Fusarium oxysporum* (Figure 6). Our results showed that plants grown in a high but non-phytotoxic Zn or Cu concentration revealed an effective defense against FA by affecting its absorption or transportation and direct mitigation in vitro and *in planta*. Cucumber plants grown with additional Zn exhibited increased tolerance to FA with a 17% increase in mitigation rate and also by inhibiting FA transport. Cucumber grown with additional Cu absorbed less FA, and mostly FA was detoxified by Cu in vitro, with a 37% increase in mitigation rate. Furthermore, additional Zn or Cu alleviates the oxidative damage of FA to cucumber plants by modification in antioxidant enzymes system, with increased antioxidant enzymes activity and decreased lipid peroxidation level. Thus, our work is consistent with the elemental defense hypothesis but further enrich develop it. Given metal ions play a role on improving plant resistance, additional studies, including investigations on *F. oxysporum* inoculation and FA inoculation, are required to fully understand the role of FA production in the pathogenicity of *F. oxysporum* and the mode of absorption and transportation of FA. These provide the theoretical basis for direct application of as protective agents in agriculture.

## 4. Materials and Methods

### 4.1. Plant Materials

Seeds of the cucumber (*Cucumis sativus* L.) cultivar “Jinchun 4”, which is susceptible to *Fusarium oxysporum*, were cultured hydroponically on modified 0.5 × strength Hoagland solution. The composition of the Hoagland solution was as follows: 2.5 mM (NH_4_)_2_SO_4_ or Ca(NO_3_)_2_, 2.5 mM K_2_SO_4_, 1.0 mM KH_2_PO_4_, 2.0 mM MgSO_4_, 35.8 µM Fe-EDTA, 57.8 µM H_3_BO_3_, 11.4 µM MnCl_2_, 0.96 µM ZnSO_4_, 0.4 µM CuSO_4_ and 0.48 µM H_2_MoO_4_. The solution was completely renewed every three days. When the plants had grown to the 3 to 4-leaf stage, seedlings were treated with Hoagland solutions containing different concentrations of FA (Sigma Co.,St.louis, MO, USA). Concentrations of 2 µg ml^−1^ FA gave the same symptoms on cucumber seedlings as inoculation with *Fusarium oxysporum* f. sp. *cucumerinum* (FOC) in a conidial suspension (10^6^ conidia mL^−1^). As a result, this concentration of FA was used as the standard for all subsequent experiments.

In the next test, cucumber seedlings were cultured hydroponically on modified 0.5 × strength Hoagland solution with different concentrations of Fe, Mn, Cu or Zn for 24 h. Then, cucumber seedlings were cultured hydroponically on modified 0.5 x strength Hoagland solution with 2 µg mL^−1^ FA and different concentrations of ions for 72 h. Each ion consists of four concentration series: Zn concentrations were 0, 9.6, 48 and 144 µM; Cu concentrations were 0, 2, 20 and 40 µM; Mn concentrations were 0, 57, 114 and 570 µM; Fe concentrations were 0, 89.5, 179 and 358 µM. These concentrations were selected based on preliminary studies that showed that they did not affect cucumber growth, but apparent differences in toxicity mitigation to FA were achieved. The seedlings which were grown in 0.5× Hoagland nutrient solution served as the control group (CK), with Zn of 0.96 µM; Cu of 0.4 µM; Mn of 11.4 µM; Fe of 35.8 µM.

### 4.2. Thermal Imaging

The thermal infrared imaging was performed as described previously [11]. The thermal images of the whole plant under different treatments were taken at 10:00 am, which was 2 h into the light period when stomata have been open. Infrared images were obtained using an infrared camera (SC620, FLIR Systems, Inc., Wilsonville, OR, USA). Digital thermograms were analyzed using the Thermal CAM Researcher Professional 2.9 software (FLIR Systems, Inc.).

### 4.3. Wilting Index

The wilting index was recorded from 0 to 72 h after FA treatment. The plants were graded for severity of wilt on a scale of 0–5 as follows: 0, the entire plant was healthy; 1, < 20%; 2, 20–40%; 3, 40–60%; 4, 60–80% and 5, 80–100% of the leaves were wilted. The wilting index was calculated using the following formula:

(1) $$\mathrm{Wilting}\;\mathrm{index}\;\left(\mathrm{WI}\right)=\frac{\sum^{\;}\left(\mathrm{wilt}\;\mathrm{grade}\;\times \;\mathrm{no}.\;\mathrm{of}\;\mathrm{plants}\;\mathrm{rated}\right)}{\left(\;\mathrm{total}\;\mathrm{no}.\;\mathrm{of}\;\mathrm{plants}\;\times \;\mathrm{the}\;\mathrm{highest}\;\mathrm{wilt}\;\mathrm{grade}\right)\;}\times 100$$

### 4.4. The Leaf and Root Membrane Injury

Leaf relative membrane injury was determined by the electrolyte leakage method as described previously [16]. Ten leaf discs (8 mm in diameter) without midribs were cut from fully expanded leaves in the same position of each plant using a stainless-steel corer and washed several times with distilled water to remove electrolytes adhering to the edge of leaf discs. The discs were infiltrated with 20 mL distilled water in glass tubes under vacuum for 20 min (distilled water, serving as the blank control). Then, the conductivity of the diffusate was directly measured using a portable conductivity meter (MP515-02, San-Xin Instrumentation, Inc., Shanghai, China). The glass tubes were subsequently boiled in a 100 °C water bath for 30 min, and a second conductivity measurement was performed after the sample cooled to 25 °C. Membrane injury was expressed as the relative injury (%), which is defined as the ratio of the first to the second conductivity readings (blank controls, subtracted). Root relative membrane injury was determined similarly as above. The 0.5 g fresh weight of the root was used for each assay.

### 4.5. Gas Exchange Measurements

The photosynthetic parameters of cucumber newly expanded leaves were measured using a portable photosynthesis open system (LI-6400; Li-Cor Biosciences, Lincoln, NE, USA). For measurement of net photosynthetic rate (Pn), stomatal conductance (gs), intercellular CO_2_ concentration (Ci) and transpiration rate (E), the leaves were maintained at a temperature of 27.8 ± 0.2°C and a relative humidity of 43.6 ± 1.3% under a photosynthetic photon flux density of 1, 200 μ mol photons m^−2^ s^−1^ during the measurements. The data were recorded after the systems reached a steady-state equilibrium (approximately 10 min).

### 4.6. Determination of Zn and Cu Concentration

Oven-dried plant tissues were finely powdered with a homogenizer (Tissuelyser-24, Shanghai Jingxin Industrial Development Co., Ltd., Shanghai, China). About 0.1 g of material was pre-digested overnight in 5 mL of 85% HNO_3_/15% HClO_4_ (*v*/*v*) and then digested during 4 h with a temperature process of 60 °C–120 °C–150 °C–190 °C. The final volume was adjusted to 25 mL with ultrapure water, and ion concentration was analyzed by inductively coupled plasma mass spectrometry (ICP-MS, NexION 300×, PerkinElmer Inc., Waltham, MA, USA).

### 4.7. FA Detection in Plants and Nutrient Solution

A method previously reported was optimized for FA extraction and analysis [73]. Harvested samples of roots, stems, leaves were ground in a homogenizer (Tissuelyser-24, Shanghai Jingxin Industrial Development Co., Ltd.) with 1:1 methanol-1% KH_2_PO_4_ (*v*/*v*) acidified to pH 2.5 with HCl and analyzed by high-performance liquid chromatography (HPLC, Agilent Technologies 1260 II LC, Santa Clara, Calif, USA). For more details, please refer to our previous studies [74,75]. The samples were quantified against a standard curve of synthetic FA (Sigma). After plants were harvested, the nutrient solution was collected and was filtrated with 0.45mm membrane filters for FA analysis with HPLC.

### 4.8. Toxicity Mitigation Rate

For the control after FA treatment, the whereabouts of FA at the end of the experiment mainly included the residual amount in the nutrient solution, the amount of absorption in cucumber plant, and lost parts due to natural degradation or other reactions. Compared to the control after FA treatment, both the high Zn and Cu treatments had a more lost portion due to the chelation reaction between FA and Zn^2+^ or Cu^2+^, or other unknown whereabouts. We refer to this part as the amount of mitigation.

### 4.9. In Vitro Toxicity Mitigation

To test the ability of FA to be detoxified by metal ions, CuSO_4_ and ZnSO_4_ were mixed in an aqueous solution of FA (2 µg mL^−1^) for 24 h at room temperature. The concentrations of Zn or Cu used varied: 8, 16, 32, 64 and 96 µM. Thereupon, the liquid was collected to determine the FA concentration in order to calculate mitigation rates.

### 4.10. Quantitative Determination of Hydrogen Peroxide, Lipid Peroxidation, Antioxidant Enzyme Activity, Lignin and Cellulose

Hydrogen peroxide (H_2_O_2_) content was measured spectrophotometrically after reaction with KI according to Alexieva et al. with some modifications [76]. The plant root or leaf tissue were homogenized in 0.1% cold trichloro acetic acid (TCA) and centrifuged at 19,000 g for 20 min at 4 °C. The 0.5 mL of supernatant was mixed with 0.5 mL of 100 mM K-phosphate buffer (pH 7.8) and 2 mL of 1 M KI (*w*/*v* in fresh double-distilled water). The absorbance was recorded at 390 nm after 1 h of incubation in darkness. The amount of H_2_O_2_ was calculated by a calibration curve prepared with known concentrations.

Lipid peroxidation was determined by quantifying the equivalents of malondialdehyde (MDA) produced by the thiobarbituric acid (TBA) reaction as described by Hodges et al. [77], and the absorbance values of the red adduct at 450, 532, and 600nm were recorded to calculate the MDA equivalents.

For the determination of antioxidant enzymes, root or leaf tissue (0.5g FW) was ground with 5 mL of pre-cold phosphate buffer (50 mM, pH 7.8) containing 1% (*w*/*v*) insoluble polyvinylpyrrolidone and 0.2 mM ethylenediaminetetraacetic acid. The homogenates were centrifuged at 12,000g for 20 min at 4 °C and the supernatants were collected for enzymatic activity analysis. Superoxide dismutase (SOD) activity was assayed by measuring the reduction of nitro blue tetrazolium (NBT) at 560 nm using the method of Stewart and Bewley [78], where one unit of SOD activity is defined as the 50% inhibition of the reduction of NBT. Catalase (CAT) and peroxidase (POD) were measured through biochemical methods, as described by Wang [79]. The CAT activity was measured by the decrease rate in the absorbance of H_2_O_2_ at 240 nm while POD activity was determined at 470 nm using the guaiacol method. The measurement of ascorbate peroxidase (APX) followed the method of Nakano and Asada [80] by the decrease in the absorbance at 290 nm.

### 4.11. Statistical Analysis

The experiments were repeated four times. A one-way analysis of variance (ANOVA) was applied to assess differences in each parameter among treatments with the SPSS Statistics 20.0 software (IBM, Chicago, IL, USA). The means and standard deviation are presented based on four independent experiments. The differences between treatments were determined using the Duncan’s multiple range tests and were indicated by different letters.

## Figures and Tables

**Table 1:** Growth and photosynthesis parameters of cucumber plants regulated by Zn and Cu under fusaric acid supply.

Treatments	CK	CK + FA	Zn + FA	Cu + FA
Plant height (cm)	18.3 ± 1.46 a	15.2 ± 0.28 b	18.7 ± 2.08 a	17.5 ± 0.50 ab
Root length (cm)	15.5 ± 1.15 a	14.7 ± 0.60 a	15.0 ± 1.70 a	14.3 ± 0.57 a
SPAD	48.9 ± 1.30 a	43.3 ± 0.60 b	49.6 ± 2.00 a	49.9 ± 1.90 a
Plant dry weight (g)	0.71 ± 0.02 b	0.62 ± 0.03 c	0.83 ± 0.02 a	0.80 ± 0.04 a
Pn(μmolCO_2_m^−2^s^−1^)	22.0 ± 0.67 a	13.8 ± 1.51 c	17.0 ± 0.16 b	18.0 ± 0.28 b
g_s_(molH_2_Om^−2^s^−1^)	0.74 ± 0.01 a	0.12 ± 0.00 c	0.48 ± 0.01ab	0.62 ± 0.02 a
Ci(μmolCO_2_mol^−1^)	357 ± 7.63 a	258 ± 5.50 c	328 ± 0.95 b	365 ± 6.35 a
E(mmolH_2_Om^−2^s^−1^)	9.06 ± 0.49 a	3.08 ± 0.29 b	9.43 ± 0.02 a	9.16 ± 0.02 a
E/g_s_(10^−3^)	7.42 ± 0.79 b	18.7 ± 1.62 a	8.42 ± 0.03 b	7.39 ± 0.11 b
Wilting index (%)	-	100 ± 5.77 a	7.3 ± 0.76 b	8.63 ± 0.30 b

Note: Plants of 3-week-old seedlings of cucumber plants were immersed in Hoagland solutions with indicated concentrations of FA (2 µg ml^−1^) for 3 days. SPAD, the leaf chlorophyll content; Plant Dry weight, the whole plant tissues; Pn, the net photosynthetic rate; g_s_, the stomatal conductance; Ci, the intercellular CO_2_ concentration; E, the transpiration rate; E/g_s_, the ratio of transpiration rate to stomatal conductance. The ratio of the transpiration rate to stomatal conductance (E/g_s_) was calculated to indicate the water loss outside of the stomata. The data were shown as the mean ± SD of four replicates. Significant differences (*p* < 0.05) among different treatments are indicated by different letters.

## Supplementary Materials

Supplementary materials can be found at https://www.mdpi.com/1422-0067/21/9/3370/s1. Figure S1. Effect of copper or zinc on the growth of cucumber seedlings in absence or presence of the FA conditions. Plants of 3-week-old seedlings of cucumber plants were immersed in Hoagland solutions with or without indicated concentrations of FA (2 µg mL^−1^) or copper or zinc for 2 days. CK, control plants receiving 0.5 × Hoagland nutrient solution, without FA; FA, 0.5 × Hoagland nutrient solution, with FA; Zn-FA, 0.5 × Hoagland nutrient solution containing Zn of 48 µM, without FA; Cu-FA, 0.5 × Hoagland nutrient solution containing Cu of 20 µM, without FA; Zn + FA, 0.5 × Hoagland nutrient solution containing Zn of 48 µM, with FA; Cu + FA, 0.5 × Hoagland nutrient solution containing Cu of 20 µM. with FA. The red arrow indicates wilting. Figure S2. Effects of Zn or Cu on the activities of H_2_O_2_, MDA and antioxidant enzyme in roots (A) and leaves (B) of cucumber plants supplemented Zn or Cu, without FA. Plants of 3-week-old seedlings of cucumber plants were immersed in Hoagland solutions with or without indicated concentrations of copper or zinc for 24 h. Bars represent standard deviation with five replications each. Different letters above bars indicate significant differences (*p* < 0.05, Duncan’s multiple range test) among different treatments.

## References

[1] Dean R., Van Kan J.A., Pretorius Z.A., Hammond-Kosack K.E., Di P.A., Spanu P.D., Rudd J.J., Dickman M., Kahmann R., Ellis J. (2012). The top 10 fungal pathogens in molecular plant pathology. Mol. Plant Pathol..

[2] Dong X., Xiong Y.F., Ling N., Shen Q.R., Guo S.W. (2014). Fusaric acid accelerates the senescence of leaf in banana when infected by *Fusarium*. World J. Microb. Biot..

[3] Niehaus E.M., Díaz-Sánchez V., Bargen K.W.V., Kleigrewe K., Humpf H.U., Limón M.C., Tudzynski B., Martín J.F., García-Estrada C. (2014). Fusarins and fusaric acid in *Fusaria*. Biosynthesis and Molecular Genetics of Fungal Secondary Metabolites.

[4] Stipanovic R.D., Puckhaber L.S., Liu J., Bell A.A. (2011). Phytotoxicity of fusaric acid and analogs to cotton. Toxicon.

[5] Venter S.L., Steyn P.J. (1998). Correlation between fusaric acid production and virulence of isolates of *Fusarium oxysporum* that causes potato dry rot in South Africa. Potato Res..

[6] Jiao J., Sun L., Zhou B., Gao Z., Yu H., Zhu X., Liang Y. (2014). Hydrogen peroxide production and mitochondrial dysfunction contribute to the fusaric acid-induced programmed cell death in tobacco cells. J. Plant Physiol..

[7] Wu H.S., Yin X.M., Liu D.Y., Ling N., Bao W., Ying R.R., Zhu Y.Y., Guo S.W., Shen Q.R. (2008). Effect of fungal fusaric acid on the root and leaf physiology of watermelon (*Citrullus lanatus*) seedlings. Plant. Soil.

[8] Samadi L., Shahsavan B.B. (2006). Fusaric acid induces apoptosis in saffron root-tip cells: Roles of caspase-like activity, cytochrome C, and H_2_O_2_. Planta.

[9] Dong X., Ling N., Wang M., Shen Q.R., Guo S.W. (2012). Fusaric acid is a crucial factor in the disturbance of leaf water imbalance in *Fusarium*-infected banana plants. Plant Physiol. Bioch..

[10] Ruiz J.A., Bernar E.M., Jung K. (2015). Production of siderophores increases resistance to fusaric acid in *Pseudomonas protegens* Pf-5. PLoS ONE.

[11] Wang M., Ling N., Dong X., Zhu J.Y., Shen Q.R., Guo S.W. (2012). Thermographic visualization of leaf response in cucumber plants infected with the soil-borne pathogen *Fusarium oxysporum* f. sp.. Cucumerinum. Plant Physiol. Bioch..

[12] Wang M., Sun Y.M., Sun G.M., Liu X., Zhai L., Shen Q.R., Guo S.W. (2015). Water balance altered in cucumber plants infected with *Fusarium oxysporum* f. sp.. Cucumerinum. Sci. Rep. UK.

[13] Czymmek K.J., Fogg M., Powell D.H., Sweigard J., Park S.Y., Kang S. (2007). In vivo time-lapse documentation using confocal and multi-photon microscopy reveals the mechanisms of invasion into the *Arabidopsis* root vascular system by *Fusarium oxysporum*. Fungal Genet. Biol..

[14] Oubraim S., Sedra M.H., Lazrek H.B. (2016). A relationship between Bayoud disease severity and toxin susceptibility of date palm cultivars. Emir. J. Food Agric..

[15] D’Alton A., Etherton B. (1984). Effects of fusaric acid on tomato root hair membrane potentials and ATP levels. Plant Physiol..

[16] Wang M., Ling N., Dong X., Liu X., Shen Q., Guo S. (2014). Effect of fusaric acid on the leaf physiology of cucumber seedlings. Eur. J. Plant Pathol..

[17] Yang S.S. (1996). Studies on cross protection of fusarium wilt of cucumber-(4)-protective effect by a nonpathogenic isolate of *Fusarium oxysporum* in a greenhouse and fields. Korean J. Plant Pathol..

[18] Boyd R. (2010). Elemental defenses of plants by metals. Nat. Educ. Knowl..

[19] Boyd R.S. (2012). Plant defense using toxic inorganic ions: Conceptual models of the defensive enhancement and joint effects hypotheses. Plant Sci..

[20] Cabot C., Martos S., Llugany M., Gallego B., Tolrà R., Poschenrieder C. (2019). A role for zinc in plant defense against pathogens and herbivores. Front. Plant Sci..

[21] Cobbett C. (2003). Heavy metals and plants-model systems and hyperaccumulators. New Phytol..

[22] Poschenrieder C., Roser T., Juan B. (2006). Can metals defend plants against biotic stress?. Trends Plant Sci..

[23] Rolke Y., Liu S., Quidde T., Williamson B., Schouten A., Weltring K.M., Siewers V., Tenberge K.B., Tudzynski B., Tudzynski P. (2004). Functional analysis of H_2_O_2_-generating systems in *B. cinerea*: The major Cu-Zn SOD (BCSOD1) has impact on virulence on bean, whereas a glucose oxidase (BCGOD1) is dispensable. Mol. Plant Pathol..

[24] Stolpe C., Giehren F., Krämer U., Müller C. (2017). Both heavy metal-amendment of soil and aphid-infestation increase Cd and Zn concentrations in phloem exudates of a metal-hyperaccumulating plant. Phytochemistry.

[25] Franza T., Mahé B., Expert D. (2005). Erwinia chrysanthemi requires a second iron transport route dependent of the siderophore achromobactin for extracellular growth and plant infection. Mol. Microbiol..

[26] Babitha M.P., Bhat S.G., Prakash H.S., Shetty H.S. (2002). Differential induction of superoxide dismutase in downy mildew-resistant and -susceptible genotypes of pearl millet. Plant Pathol..

[27] Parisot D., Dufresne M., Veneault C., Laugé R., Langin T. (2002). Clap1, a gene encoding a copper-transporting ATPase involved in the process of infection by the phytopathogenic fungus *Colletotrichum lindemuthianum*. Mol. Genet. Genom..

[28] Fones H., Davis C.A.R., Rico A., Fang F., Smith J.A.C., Preston G.M. (2010). Metal hyperaccumulation armors plants against disease. PLoS Pathog..

[29] Fones H., Preston G.M. (2013). The impact of transition metals on bacterial plant disease. FEMS Microbiol. Rev..

[30] Gallego B., Martos S., Cabot C., Barceló J., Poschenrieder C. (2017). Zinc hyperaccumulation substitutes for defense failures beyond salicylate and jasmonate signaling pathways of *Alternaria brassicicola* attack in *Noccaea caerulescens*. Physiol. Plant..

[31] Heckman J.R., Clarke B.B., Murphy J.A. (2003). Optimizing manganese fertilization for the suppression of take-all patch disease on creeping bentgrass. Crop Sci..

[32] Simoglou K.B., Dordas C. (2006). Effect of foliar applied boron, manganese and zinc on tan spot in winter durum wheat. Crop Prot..

[33] Mithöfer A., Schulze B., Boland W. (2004). Biotic and heavy metal stress response in plants: Evidence for common signals. FEBS Lett..

[34] Martos S., Gallego B., Cabot C., Llugany M., Barceló J., Poschenrieder C. (2016). Zinc triggers signaling mechanisms and defense responses promoting resistance to *Alternaria brassicicola* in *Arabidopsis thaliana*. Plant Sci..

[35] Freeman J.L., Garcia D., Kim D., Hopf A., Salt D.E. (2005). Constitutively elevated salicylic acid signals glutathione-mediated nickel tolerance in *Thlaspi* nickel hyperaccumulators. Plant Physiol..

[36] Jonak C., Nakagami H., Hirt H. (2004). Heavy metal stress. Activation of distinct mitogen-activated protein kinase pathways by copper and cadmium. Plant Physiol..

[37] Mittra B., Ghosh P., Henry S.L., Mishra J., Das T.K., Ghosh S., Babu C.R., Mohanty P. (2004). Novel mode of resistance to *Fusarium* infection by a mild dose pre-exposure of cadmium in wheat. Plant Physiol. Biochem..

[38] Malini S. (2010). Heavy metal chelates of fusaric acid: In vitro spectrophotometry. J. Phytopathol..

[39] Pan J.H., Lin Y.C., Ni T., Gu Y.C. (2010). Cu (II): A “signaling molecule” of the mangrove endophyte *Fusarium oxysporum* ZZF51?. Biometals.

[40] Lakshminarayanan K., Subramanian D. (1955). Is fusaric acid a vivotoxin?. Nature.

[41] Tamari K., Kaji J. (1952). Studies on the mechanism of injurious action of fusarinic acid on plant growth. J. Agric. Chem. Soc. Jpn..

[42] Yin E.S., Rakhmankulova M., Kucera K., Filho J.G.D.S., Portero C.E., Narváez-Trujillo A., Holley S.A., Strobel S.A. (2015). Fusaric acid induces a notochord malformation in zebrafish via copper chelation. Biometals.

[43] López-Díaz C., Rahjoo V., Sulyok M., Ghionna V., Martín-Vicente A., Capilla J., Di P.A., López-Berges M.S. (2017). Fusaric acid contributes to virulence of *Fusarium oxysporum* on plant and mammalian hosts. Mol. Plant Pathol..

[44] Singh V.K., Singh H.B., Upadhyay R.S. (2017). Role of fusaric acid in the development of ’*Fusarium* wilt’ symptoms in tomato: Physiological, biochemical and proteomic perspectives. Plant Physiol. Biochem..

[45] Chawla H.S., Wenzel G. (1987). In vitroselection for fusaric acid resistant barley plants. Plant Breed..

[46] Jiang R.F., Ma D.Y., Zhao F.J., Mcgrath S.P. (2010). Cadmium hyperaccumulation protects *Thlaspi caerulescens* from leaf feeding damage by thrips (*Frankliniella occidentalis*). New Phytol..

[47] Behmer S.T., Lloyd C.M., Raubenheimer D., Stewart-Clark J., Knight J., Leighton R.S., Harper F.A., Smith J.A.C. (2010). Metal hyperaccumulation in plants: Mechanisms of defence against insect herbivores. Funct. Ecol..

[48] Ent A.V.D., Baker A.J.M., Reeves R.D., Pollard A.J., Schat H. (2013). Hyperaccumulators of metal and metalloid trace elements: Facts and fiction. Plant Soil.

[49] Reeves R.D. (2003). Tropical hyperaccumulators of metals and their potential for phytoextraction. Plant Soil.

[50] Saikia R., Varghese S., Singh B.P., Arora D.K. (2009). Influence of mineral amendment on disease suppressive activity of *Pseudomonas fluorescens* to *Fusarium* wilt of chickpea. Microbiol. Res..

[51] Marrè M.T., Vergani P., Albergoni F.G. (1993). Relationship between fusaric acid uptake and its binding to cell structures by leaves of *Egeria densa* and its toxic effects on membrane permeability and respiration. Physiol. Mol. Plant Pathol..

[52] Pavlovkin J., Mistrík I., Prokop M. (2004). Some aspects of the phytotoxic action of fusaric acid on primary *Ricinus* roots. Plant Soil Environ..

[53] Vázquez M.D., Poschenrieder C., Barceló J., Baker A.J.M., Hatton P., Cope G.H. (1994). Compartmentation of zinc in roots and leaves of the zinc hyperaccumulator *Thlaspi caerulescens* J. C. Presl. Bot. Acta.

[54] Schneider T., Persson D.P., Husted S., Schellenberg M., Gehrig P., Lee Y., Martinoia E., Schjoerring J.K., Meyer S. (2013). A proteomics approach to investigate the process of Zn hyperaccumulation in *Noccaea caerulescens* (J & C. Presl) F.K. Meyer. Plant J..

[55] Peng J., Gong J. (2014). Vacuolar sequestration capacity and long-distance metal transport in plants. Front. Plant Sci..

[56] Lu L., Tian S., Zhang J., Yang X., Labavitch J.M., Webb S.M., Latimer M., Brown P.H. (2013). Efficient xylem transport and phloem remobilization of Zn in the hyperaccumulator plant species S edum alfredii. New Phytol..

[57] Álvarez-Fernández A., Díaz-Benito P., Abadía A., López-Millán A.-F., Abadía J. (2014). Metal species involved in long distance metal transport in plants. Front. Plant Sci..

[58] Pilon M., Abdelghany S.E., Cohu C.M., Gogolin K.A., Ye H. (2006). Copper cofactor delivery in plant cells. Curr. Opin. Plant Biol..

[59] Claudia C., Enrico M., Catherine K. (2004). Hyperaccumulation of cadmium and zinc in *Thlaspi caerulescens* and *Arabidopsis halleri* at the leaf cellular level. Plant Physiol..

[60] Tan N., Pan J.H., Peng G.T., Mou C.B., Tao Y.W., She Z.G., Yang Z.L., Zhou S.N., Lin Y.C. (2008). A copper coordination compound produced by a marine fungus *Fusarium* sp. ZZF51 with biosorption of Cu (II) ions. Chin. J. Chem..

[61] Jian Y., Meredith M., Stack B.C. (2013). Effects of fusaric acid treatment on HEp2 and docetaxel-resistant HEp2 laryngeal squamous cell carcinoma. Chemotherapy.

[62] Devaraja S., Girish K.S., Santhosh M.S., Hemshekhar M., Nayaka S.C., Kemparaju K. (2013). Fusaric acid, a mycotoxin, and its influence on blood coagulation and platelet function. Blood Coagul. Fibrinolysis.

[63] Krämer U. (2010). Metal hyperaccumulation in plants. Annu. Rev. Plant Biol..

[64] Noman A., Aqeel M., Khalid N., Islam W., Sanaullah T., Anwar M., Khan S., Ye W., Lou Y. (2019). Zinc finger protein transcription factors: Integrated line of action for plant antimicrobial activity. Microb. Pathog..

[65] Yruela I. (2009). Copper in plants: Acquisition, transport and interactions. Funct. Plant Biol..

[66] Hänsch R., Mendel R.R. (2009). Physiological functions of mineral micronutrients (Cu, Zn, Mn, Fe, Ni, Mo, B., Cl). Curr. Opin. Plant Biol..

[67] Hall J.L. (2002). Cellular mechanisms for heavy metal detoxification and tolerance. J. Exp. Bot..

[68] Cakmak I. (2010). Tansley review no. 111: Possible roles of zinc in protecting plant cells from damage by reactive oxygen species. New Phytol..

[69] Singh V.K., Upadhyay R. Induction of defence responses by fusaric acid (*Fusarium* toxin) in tomato plant. Proceedings of the 6th International Conference on Agriculture, Environment and Biological Sciences.

[70] Broadley M., Brown P., Cakmak I., Rengel Z., Zhao F.J. (2012). Function of nutrients: Micronutrients. Marschner’s Mineral Nutrition of Higher Plants.

[71] Li J., Essemine J., Shang C., Zhang H., Zhu X., Yu J., Chen G., Qu M., Sun D. (2020). Combined proteomics and metabolism analysis unravels prominent roles of antioxidant system in the prevention of alfalfa (*Medicago sativa* L.) against salt stress. Int. J. Mol. Sci..

[72] Ahmad R.M., Cheng C., Sheng J., Wang W., Ren H., Aslam M., Yan Y. (2019). Interruption of jasmonic acid biosynthesis causes differential responses in the roots and shoots of maize seedlings against salt stress. Int. J. Mol. Sci..

[73] Smith T.K., Sousadias M.G. (1994). Fusaric acid content of swine feedstuffs. J. Agric. Food Chem..

[74] Zhou J., Wang M., Sun Y., Gu Z., Wang R., Saydin A., Shen Q., Guo S. (2017). Nitrate increased cucumber tolerance to *Fusarium* wilt by regulating fungal toxin production and distribution. Toxins.

[75] Wang M., Gu Z.C., Wang R.R., Guo J.J., Ling N., Firbank L.G., Guo S.W. (2019). Plant primary metabolism regulated by nitrogen contributes to plant-pathogen interactions. Plant Cell Physiol..

[76] Alexieva V., Sergiev I., Mapelli S., Karanov E. (2001). The effect of drought and ultraviolet radiation on growth and stress markers in pea and wheat. Plant Cell Environ..

[77] Hodges D.M., DeLong J.M., Forney C.F., Prange R.K. (1999). Improving the thiobarbituric acid-reactive-substances assay for estimating lipid peroxidation in plant tissues containing anthocyanin and other interfering compounds. Planta.

[78] Stewart R.R.C., Bewley J.D. (1980). Lipid peroxidation associated with accelerated aging of soybean axes. Plant Physiol..

[79] Wang X., Wang X. (2006). Plant Physiology and Biochemistry Experiment Principle and Technology.

[80] Nakano Y., Asada K. (1981). Hydrogen peroxide is scavenged by ascorbate-specific peroxidase in spinach chloroplasts. Plant Cell Physiol..

