# Supplementary material for: Zinc and Copper Enhance Cucumber Tolerance to Fusaric Acid by Mediating Its Distribution and Toxicity and Modifying the Antioxidant System
Source: Int J Mol Sci. 2020 May 10;21(9):3370. doi: 10.3390/ijms21093370 (PMC7247006; doi:10.3390/ijms21093370)
Supplement: Supplementary file 1 [file ijms-21-03370-s001.pdf]

Supplementary Materials:

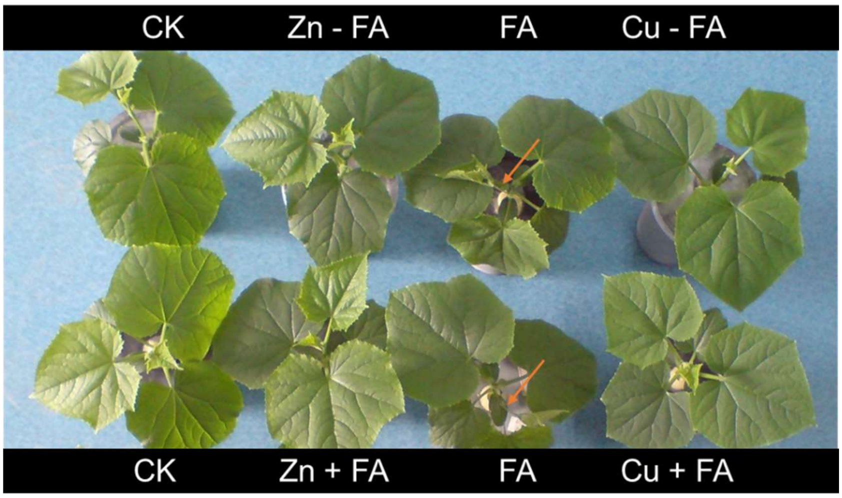

Figure S1. Effect of copper or zinc on the growth of cucumber seedlings in absence or presence of the FA conditions. Plants of 3-week-old seedlings of cucumber plants were immersed in Hoagland solutions with or without indicated concentrations of FA (2 μg ml^{-1}) or copper or zinc for 2 days. CK, control plants receiving 0.5 × Hoagland nutrient solution, without FA; FA, 0.5 × Hoagland nutrient solution, with FA; Zn-FA, 0.5 × Hoagland nutrient solution containing Zn of 48 μM, without FA; Cu-FA, 0.5 × Hoagland nutrient solution containing Cu of 20 μM, without FA; Zn + FA, 0.5 × Hoagland nutrient solution containing Zn of 48 μM, with FA; Cu + FA, 0.5 × Hoagland nutrient solution containing Cu of 20 μM. with FA. The red arrow indicates wilting.

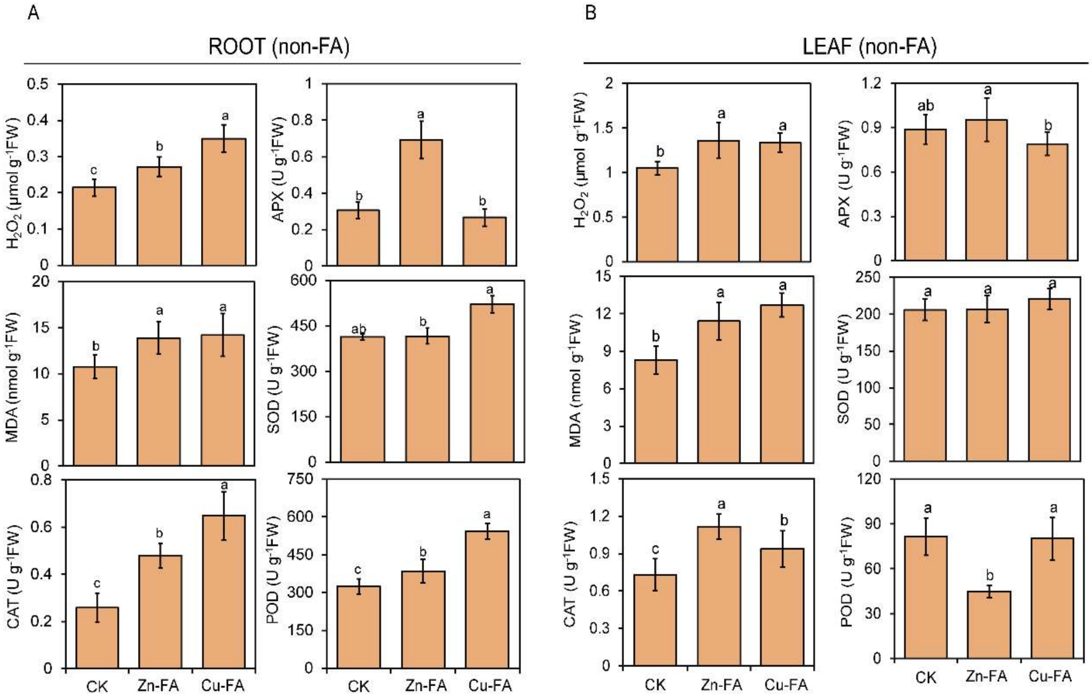

Figure S2. Effects of Zn or Cu on the activities of H2O2, MDA and antioxidant enzyme in roots (**A**) and leaves (**B**) of cucumber plants supplemented Zn or Cu, without FA. Plants of 3-week-old seedlings of cucumber plants were immersed in Hoagland solutions with or without indicated concentrations of copper or zinc for 24 h. Bars represent standard deviation with five replications each. Different letters above bars indicate significant differences ($p < 0.05$, Duncan's multiple range test) among different treatments.
